# Randomized trial of transcutaneous tibial nerve stimulation to treat overactive bladder in older women

**DOI:** 10.1371/journal.pone.0322508

**Published:** 2026-04-06

**Authors:** Marianna Vale D’Alessandro Barbosa, Liana Barbaresco Gomide Matheus, Patrícia Azevedo Garcia, Júlia Shimohara Bradaschia, Marianne Lucena da Silva, Elaine Cristina Leite Pereira, Aline Teixeira Alves

**Affiliations:** 1 Faculdade de Ciências e Tecnologias em Saúde, Programa de Pós-Graduação em Ciências da Reabilitação, Universidade de Brasilia, Brasília, Distrito Federal, Brasil; 2 Faculdade de Ciências e Tecnologias em Saúde, Universidade de Brasília, Brasília, Distrito Federal, Brasil; 3 Departamento de Saúde Coletiva, Universidade de Brasília, Brasília, Distrito Federal, Brasil; China Medical University, TAIWAN

## Abstract

**Objectives:**

To evaluate the effects of transcutaneous tibial nerve stimulation (TTNS) associated with behavior therapy (BT) compared to BT alone in the treatment of Overactive Bladder Syndrome (OAB) in older women.

**Study design:**

Randomized controlled clinical trial in two groups, G1 that received BT (n = 19) and G2 with the addition of TTNS (n = 19).

**Main outcome measures:**

The variables analyzed were impact on quality of live (QOL) and degree of discomfort of the symptoms of OAB by International Consultation on Incontinence Questionnaire Overactive Bladder (ICIQ-OAB) and voiding habit by a 3-day voiding diary (VD). The assessments were conducted at the beginning and the end of the treatment.

**Results:**

Both groups showed a significant reduction in the impact of QOL by ICIQ-OAB. The G1 (BT) reduced the discomfort of nocturia symptoms and urgency urinary incontinence, while in G2 (BT+TTNS), it just did not present a reduction in the discomfort of urinary frequency by ICIQ-OAB. In the VD, despite G1 presenting an episode reduction of urgency urinary incontinence and nocturia, it showed no significant difference. In the G2, the same variables had significant reduction.

**Conclusion:**

Behavioral therapy reduced OAB symptoms and discomfort in older women, but its combination with TTNS led to greater improvements, particularly in urgency and nocturia. TTNS was safe, well tolerated, and enhanced quality of life, although further studies with larger samples and longer follow-up are warranted. No adverse events or complications were observed, supporting the safety and tolerability of TTNS in older women with OAB.

## Introduction

Overactive Bladder Syndrome (OAB), according to the International Continence Society (ICS), is a clinical diagnostics characterized by the presence of urinary urgency, usually accompanied by increased frequency and nocturia, with or without urgency urinary incontinence, in absence of urinary tract infection or other obvious pathologies [1]. Women are more likely to experience discomfort with the symptoms of OAB, and the more frequent these are, the more severe is their discomfort [2].

Its global prevalence is high in both sexes [3] and tends to increase with age [2]. In Brazil, a multicenter study of prevalence found the presence of symptoms of OAB in 27.8% of men and 21.3% of women [4] in a group aged above 70 years old.

The aging process causes physiological changes from the brain to the bladder, with a decrease in the neural control of continence, increase activation areas responsible for the urge to urinate, increase spontaneous contractions in the bladder, in addition to anatomical and hormonal changes that contribute to the development of micturition symptoms [5,6].

OAB harms patients’ quality of life (QOL) [7] and is related to increased levels of anxiety and depression [8]. The impact on sexual function and the impact on productivity at work favors the social isolation of the elderly [9] It increases the risk of falls and fractures in the elderly, which can lead to death [10].

Among the treatment options, the guidelines [11,12] suggest, as the gold standard, initially conservative treatment with behavior therapy (BT). The BT consists of bladder training, pelvic floor muscle training (PFMT), and fluid intake control. The second line recommends using drugs that include antimuscarinic agents and agonists β3 adrenoceptors that can be administered alone or combined. In third-line therapy, there is transcutaneous and percutaneous electrostimulation in the tibial and sacral nerves, indicated for refractory patients [11,12].

Transcutaneous tibial nerve stimulation (TTNS) has been increasingly studied in the management of OAB. It is a non-invasive, effective, safe, and tolerable treatment for reducing urinary symptoms in both adults and the elderly, with idiopathic and neurogenic OAB [13]. A study suggested the use of TTNS associated with BT as a first-line treatment for the treatment of OAB symptoms in older women [14].

The high prevalence of OAB and the possible comorbidities that can complicate the use of medications and other invasive procedures, in addition to the restriction that some women experience vaginal manipulation, makes the elderly population a group of special interest for the application of TTNS. We hypothesize that TTNS may be an effective treatment as a conservative treatment for older women with OAB.

## Materials and methods

A randomized and controlled trial was carried out, with a blind evaluator and a comparison between control and intervention groups, conducted at the Health Center nº. 4 in the administrative region of Ceilândia – located in the Federal District, Brazil. Participants were recruited in the follow-up period from January 2017 to January 2022.

All participants were informed and signed the consent form before the study started. The study was conducted in agreement with ethical standards and has the informed consent of the participants in accordance with National Health Council (NHC) Resolution 196/96 of the Brazilian Ministry of Health, which oversees the Human Research Ethics Code. This clinical trial was registered in the Rebec (Registros Brasileiros de Ensaios Clínicos), with the number RBR-9q7j7y.

The study inclusion criteria were older women, aged between 60 and 80 years old. We excluded women older than 80 years to avoid the confounders not controlled by initial assessment. Participants were included if they presented with symptoms of overactive bladder, as identified by a score greater than or equal to 8 on the *Overactive Bladder Questionnaire version 8* (OAB-V8) [15]. The exclusion criteria were older women with lower urinary tract infection, history of treatment for OAB in the last 6 months, underlying neurological diseases (multiple sclerosis, stroke, Parkinson’s disease, Alzheimer’s disease), history of genitourinary neoplasm, previous pelvic irradiation, vaginal prolapse that surpassed the vaginal ostium, cardiac pacemaker or when using OAB medication.

All evaluations were performed before and after the intervention by a blind physiotherapist in the study who was unaware of the participants’ allocation and did not participate in therapy.

After signing the consent form, the patients were randomized according to the randomization list generated by the website https://www.random.org/ and were divided into a behavior therapy group (G1), which included only guidelines with lifestyle changes; and an transcutaneous tibial nerve stimulation and behavior therapy (G2), which consisted of the overlap of the two treatments. The randomization and allocation of participants were done blindly. An unblinded application assistant, not involved in other study procedures, opened sealed, opaque envelopes to maintain allocation concealment.

Assessments and interventions were carried out in three stages:

### 2.1. Initial assessment

The following clinical and sociodemographic variables were collected for sample characterization: age, body mass index (BMI), obstetric history, education, ethnicity, marital status, systemic arterial hypertension (SAH), and diabetes. Physical examination, which included functional evaluation of the pelvic floor, using the PERFECT [16] scheme and evaluation of genital prolapses, using the Baden-Walker scale [17].

The degree of discomfort of the symptoms of OAB and its impact on QOL was assessed by the questionnaire International Consultation on Incontinence Questionnaire Overactive Bladder (ICIQ-OAB), which comes from the ICIQ class of the International Continence Society, translated and validated, and effective to assess the degree of discomfort of the symptoms of OAB and its impact on the quality of life. It consists of 4 questions concerning the symptoms of urinary urgency, urgency urinary incontinence, urinary frequency, and nocturia in the last 4 weeks, with a score of 0–4 for each item, and how much each symptom bothers the life of the patient, with a score ranging from 0 to 10, with 0 being no nuisance and 10 being the maximum nuisance [18].

The voiding habit was analyzed using the voiding diary (VD) for three days, with records of urinary frequency in 24 hours, nocturia, urinary urgency, and urgency urinary incontinence. The average of episodes over the 3 days was considered the final result. We opted for a diary model in the form of a pictogram to avoid losses due to possible illiteracy.

### 2.2. Intervention

Behavior therapy (BT), which was applied to both groups (G1 and G2), consisted of 2 orientation sessions, once a week, lasting 15 minutes each, individually passed by another physical therapist from the intervention team. A booklet was given with some recommendations for content fixation at the end of the session: a) Proper positioning for urination: always seated, with legs apart, torso bent forward, elbows supported on the knees, and use of firm support for the feet to keep the hip flexed above 90º; b) Programmed urination: patients were instructed to delay urination to the maximum, trying to reach a minimum interval of 2 hours; c) Programmed water intake: drink 2 liters of water well-spaced throughout the day and avoid drinking 2–3 hours before sleeping to avoid nocturia episodes; d) Control of irritating foods: avoid eating irritating foods and drinks for the bladder (such as caffeine, teas with caffeine, and sodas with caffeine).

The G2 received, in addition to BT, the TTNS performed with a Dualpex 961 device from the Quark®. The adopted procedure followed the protocol of Amarenco et al. [19]. The positive carbon electrode (size 3cmx5 cm) had its surface covered with gel. It was positioned and fixed 4 fingers above the medial malleolus, posterior to the tibia, and the negative carbon electrode (size 3cmx5 cm), mobile, was positioned in the region posterior to the medial malleoli, following the path of the tibial nerve. The correct position of the electrodes was determined by visualizing rhythmic flexions of the toes during stimulation with a frequency of 1 Hz and a pulse width of 200 µs. After fixing the electrode, the intensity was decreased, and the stimulation frequency increased to 10 Hz. The sessions lasted 30 minutes, and the intensity was the maximum tolerated by the patients, as long as it did not cause pain and discomfort. The patients were positioned with the right leg extended and supported in a chair, and the electrostimulation was applied to the right leg throughout the treatment. The sessions were held twice a week, for 4 weeks, with an interval of 48 hours, totaling 8 sessions.

The TTNS and BT were carried out for a trained team. The BT was carried out for 3 people that were not involved with the TTNS. All of them were PT (physiotherapist) with more than 3 years of experience in Women’s Health. The TTNS was carried out for a team of students in a physiotherapy course. All of them had previous training to follow the protocol, and they were supervised by a PT that was not involved with the BT.

### 2.3. Final assessment

After 4 weeks, all patients were re-evaluated using the same procedures from the initial evaluation.

The sample calculation was based on a pilot study previously carried out with 19 participants, 8 older women in the G1 group (behavior therapy) and 11 older women in the G2 group (transcutaneous tibial nerve stimulation associated with behavior therapy). For that, G-Power 3.1.9.2 program was used. The level of significance considered was 5%, and the error of 20%. Using the main outcome of the OAB symptoms’ impact on QOL through the score of the ICIQ-OAB questionnaire, a sample of 17 participants in each group was obtained.

Statistical analyzes were performed with the aid of SPSS software (26.0). The characterization of the sample profile was performed employing absolute frequency (n); relative frequency (%) for categorical variables and mean, and standard deviation for continuous variables. The normality of the data was verified using the Shapiro-Wilk test. The homogeneity of the sample profile between the groups was tested by applying the tests t by Student, Mann-Whitney U, and Chi-square by Pearson and Fisher’s exact test. The comparison of OAB symptoms before and after the intragroup intervention was analyzed using the Wilcoxon test, and the analysis between groups was used the Mann-Whitney U test. Spearman’s correlation was applied to verify the correlation between the variation observed in the degree of discomfort and voiding habit (VD) before and after the groups’ intervention. The level of significance adopted was 5% (p < 0.05). The intention-to-treat analysis was performed to preserve randomization.

## Results

**Fig 1:**
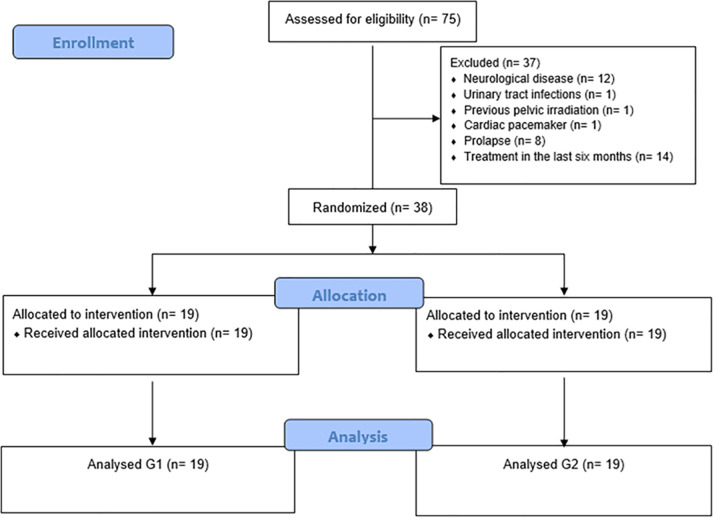
Consort flow diagram 2010.

For this study, it was considered eligible and selected for convenience, 75 older women with OAB. After the initial interview, 37 patients were excluded: 12 for neurological illnesses of the base (1 multiple sclerosis, 8 stroke, 2 Parkinson’s disease, 1 Alzheimer’s disease), 1 for infection of the lower urinary tract, 1 for previous pelvic irradiation, 1 for possessing pacemaker, 8 for vaginal prolapse, and 14 for treatment for OAB (7 of them had carried out a physiotherapeutic treatment for at least 6 months, and 7 of them for going through drug treatment for the OAB). Thus, 38 patients were randomized and composed the final sample of the study, 19 in G1 and 19 in G2, as demonstrated in the Consort Flow Diagram 2010 (Fig 1). We did not have any one participant that refused to participate in the study.

Both groups were homogeneous for the clinical characteristics, and complaints analyzed pre-treatment, as seen in Tables 1 and 2.

**Table 1:** Sociodemographic and clinical characteristics of women in G1 and G2 groups.

Average ± SD	Groups		Total (n = 38)	*p*
	G1 (n = 19)	G2 (n = 19)		
Age	71.89 ± 6.92	69.89 ± 8.01	70.89 ± 7.45	0.24^b^
Weight	70.85 ± 10.32	72.90 ± 9.10	71.88 ± 9.65	0.52^a^
Height	1.53 ± 0.08	1.54 ± 0.07	1.54 ± 0.07	0.81^a^
BMI	30.43 ± 5.72	31.02 ± 4.76	30.72 ± 5.20	0.73^a^
Pregnancies	5.89 ± 3.41	4.74 ± 2.62	5.32 ± 3.06	0.24^a^
Vaginal delivery	4.26 ± 3.46	3.26 ± 2.38	3.76 ± 2.97	0.35^b^
*n (%)*				
**Education**				
Illiterate	4 (21.1)	1 (5.3)	5 (13.2)	0.06^c^
1 to 4 years	10 (52.6)	4 (21.1)	14 (36.8)	
5 to 8 years	2 (10.5)	8 (42.1)	10 (26.3)	
High school	3 (15.8)	5 (26.3)	8 (21.1)	
University educated	0 (0.0)	1 (5.3)	1 (2.6)	
**Marital Status**				
Single	3 (15.8)	0 (0.0)	3 (7.9)	0.22^c^
Married	4 (21.1)	8 (42.1)	12 (31.6)	
Divorced	5 (26.3)	5 (26.3)	10 (26.3)	
Widow	7 (36.8)	6 (31.6)	13 (34.2)	
**Ethnicity**				
White	8 (42.1)	10 (52.6)	18 (47.4)	0.78^c^
Black	2 (10.5)	1 (5.3)	3 (7.9)	
Mulatto	3 (15.8)	1 (5.3)	4 (10.5)	
Yellow	2 (10.5)	3 (15.8)	5 (13.2)	
Other	4 (21.1)	4 (21.1)	8 (21.1)	
**Diabetes**				
Yes	3 (15.8)	4 (21.1)	7 (18.4)	0.67^c^
No	16 (84.2)	15 (78.9)	31 (81.6)	
**SAH**				
Yes	15 (78.9)	13 (68.4)	28 (73.7)	0.46^c^
No	4 (21.1)	6 (31.6)	10 (26.3)	
**Smoker**				
Yes	1 (5.3)	1 (5.3)	2 (5.3)	0.98^c^
No	18 (94.7)	18 (94.7)	36 (94.7)	

a Student’s t-test; ^b^Mann-Whitney; ^c^Pearson Chi-square.

n, absolute frequency; %, relative frequency; SD = standard deviation; SAH: systemic arterial hypertension; G1: Behavior therapy (BT); G2: BT + Transcutaneous tibial nerve stimulations (TTNS).

**Table 2:** Degree of discomfort (ICIQ-OAB) and Voiding Diary (VD) measures in women from G1 and G2 groups.

Average ± SD	Groups		Total (n = 38)	*p*
	G1 (n = 19)	G2 (n = 19)		
ICIQ-OAB (total score)	9.58 ± 3.91	8.74 ± 3.25	9.16 ± 3.57	0.47^a^
ICIQ-OAB: Degree of discomfort				
Frequency	5.74 ± 4.39	6.95 ± 3.81	6.34 ± 4.10	0.40^b^
Nocturia	7.47 ± 4.14	6.00 ± 4.29	6.74 ± 4.23	0.17^b^
Urgency	8.11 ± 2.87	8.26 ± 2.60	8.18 ± 2.70	0.98^b^
Urgency urinary incontinence	7.95 ± 3.26	7.58 ± 3.53	7.76 ± 3.36	0.76^b^
**VD**				
Frequency	7.37 ± 3.11	6.58 ± 2.58	6.98 ± 2.85	0.40^a^
Urgency	1.41 ± 2.12	1.33 ± 1.11	1.37 ± 1.67	0.31^b^
Urgency urinary incontinence	2.01 ± 2.39	1.85 ± 1.91	1.93 ± 2.14	0.71^b^
Nocturia	2.03 ± 1.59	2.24 ± 1.75	2.14 ± 1.65	0.70^a^

^to^Student’s t-test; ^b^Mann-Whitney; SD = standard deviation.

VD: voiding diary; G1: Behavior therapy (BT); G2: BT + Transcutaneous tibial nerve stimulations (TTNS).

Before treatment, the symptom of greater discomfort in both groups G1 and G2 was the voiding urgency, presenting averages by ICIQ-OAB of 8.11 ± 2.87 and 8.26 ± 2.60, respectively.

After the 4 weeks of intervention, in the intragroup comparison, G1 showed significant improvement for the QOL impact variables (total ICIQ-OAB), nocturia discomfort, and urgency urinary incontinence discomfort, but did not show significant improvement in any of the 3-day VD variables. G2 showed significant improvement in the variables analyzed by ICIQ-OAB, except for the discomfort of urinary frequency and significant reduction for episodes of nocturia and urgency urinary incontinence.

Comparing both groups (pre and post-treatment), there was a significant difference only for the urgency episodes evaluated by the 3-day VD (Table 3).

**Table 3:** Comparison of overactive bladder (OAB) symptoms before and after intervention in G1 and G2 groups.

	G1		Δ	G2		Δ	*p* ^*a*^	*p* ^*b*^	p*
	Pre	Post	Average - IC95%	Pre	Post	Average - IC95%			
ICIQ-OAB (total score)	9,58 ± 3,91	7,26 ± 4,54	−2.32 (−3.93 to −0.70)	8,74 ± 3,25	4,99 ± 2,27	−3.75 (−5.20 to −2.29)	**0,01**	**<0,001**	0,12
ICIQ-OAB: degree of discomfort									
Frequency	5,74 ± 4,39	4,53 ± 3,92	−1.20 (−2.73 to 0.33)	6,95 ± 3,81	4,96 ± 3,21	−1.98 (−4.23 to 0.27)	0,10	0,10	0,40
Nocturia	7,47 ± 4,14	4,49 ± 3,59	−2.98 (−5.17 to −0.79)	6,00 ± 4,29	2,93 ± 3,23	−3.07 (−4.77 to −1.37)	**0,01**	**0,004**	0,92
Urgency	8,11 ± 2,87	6,56 ± 3,94	−1.54 (−3.47 to 0.38)	8,26 ± 2,60	4,42 ± 3,49	−3.84 (−5.80 to −1.88)	0,18	**0,002**	0,06
Urgency urinary incontinence	7,95 ± 3,26	5,85 ± 4,00	−2.10 (−3.95 to −0.25)	7,58 ± 3,53	4,60 ± 3,16	−2.98 (−5.63 to −0.33)	**0,03**	**0,03**	0,39
**VD**									
Frequency	7,37 ± 3,11	7,53 ± 4,15	0.16 (−1.25 to 1.58)	6,58 ± 2,58	6,78 ± 1,98	0.20 (−0.64 to 1.03)	0,98	0,48	0,45
Urgency	1,41 ± 2,12	1,73 ± 2,45	0.32 (−0.98 to 1.62)	1,33 ± 1,11	1,09 ± 0,86	−0.24 (−0.78 to 0.30)	0,61	0,12	**0,01**
Urgency urinary incontinence	2,01 ± 2,39	1,48 ± 1,67	−0.53 (−1.37 to 0.32)	1,85 ± 1,91	1,06 ± 1,39	−0.79 (−1.49 to −0.09)	0,28	**0,01**	0,34
Nocturia	2,03 ± 1,59	1,95 ± 0,97	−0.08 (−0.75 to 0.58)	2,24 ± 1,75	1,62 ± 1,51	−0.62 (−1.02 to −0.23)	0,98	**0,007**	0,06

*p*^*a*^:comparison intragroup G1 (pre/post treatment); *p*^*b*^: comparison intragroup G2 (pre/post treatment) p*: comparison between groups (pre/post treatment).

Descriptive statistics Average ± standard deviation or average and 95% confidence interval.

VD: voiding diary; G1: Behavior therapy (BT); G2: BT + Transcutaneous tibial nerve stimulations (TTNS).

**Fig 2:**
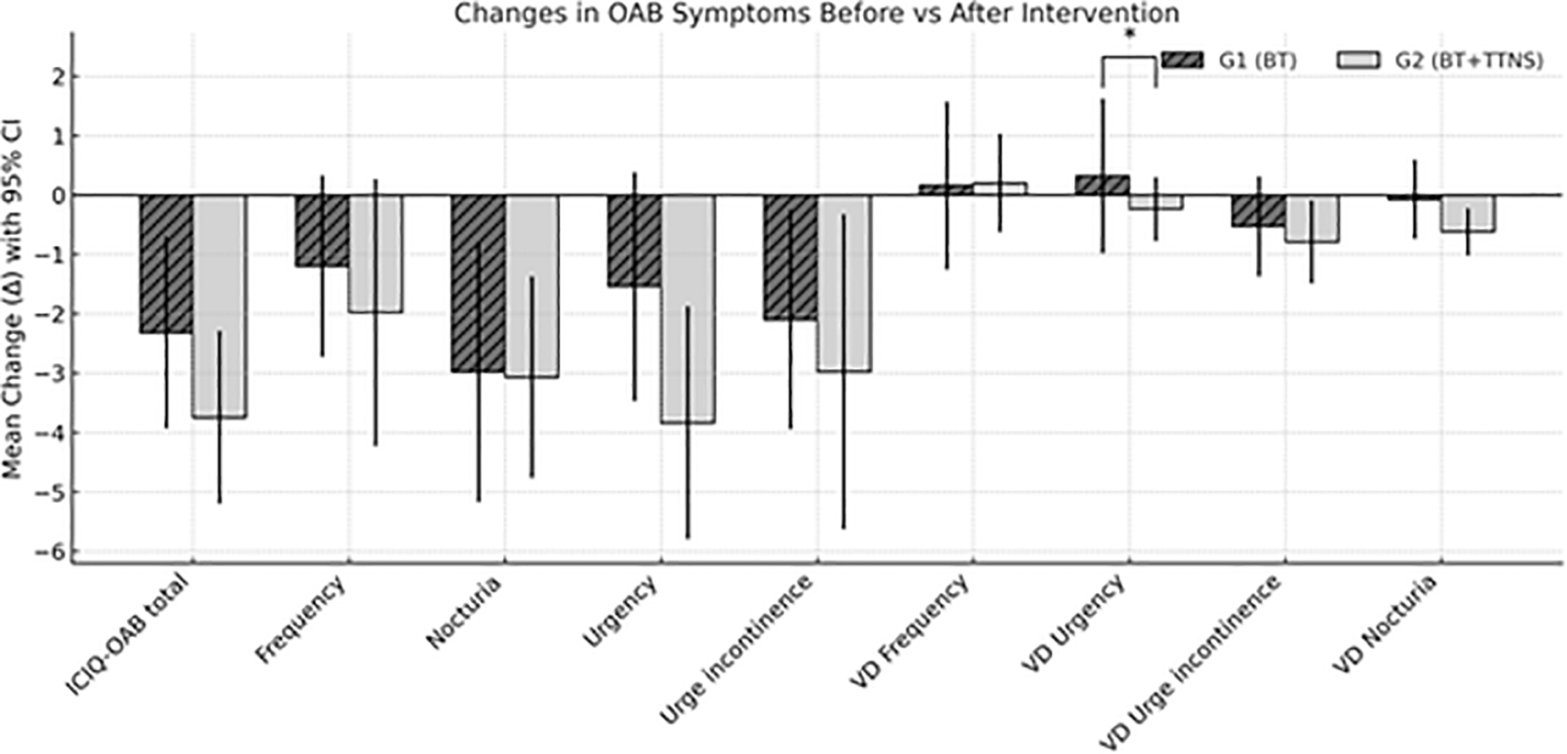
Mean change (Δ) with 95% confidence interval in overactive bladder (OAB) symptoms before and after intervention in G1 and G2 groups.

**Fig 3:**
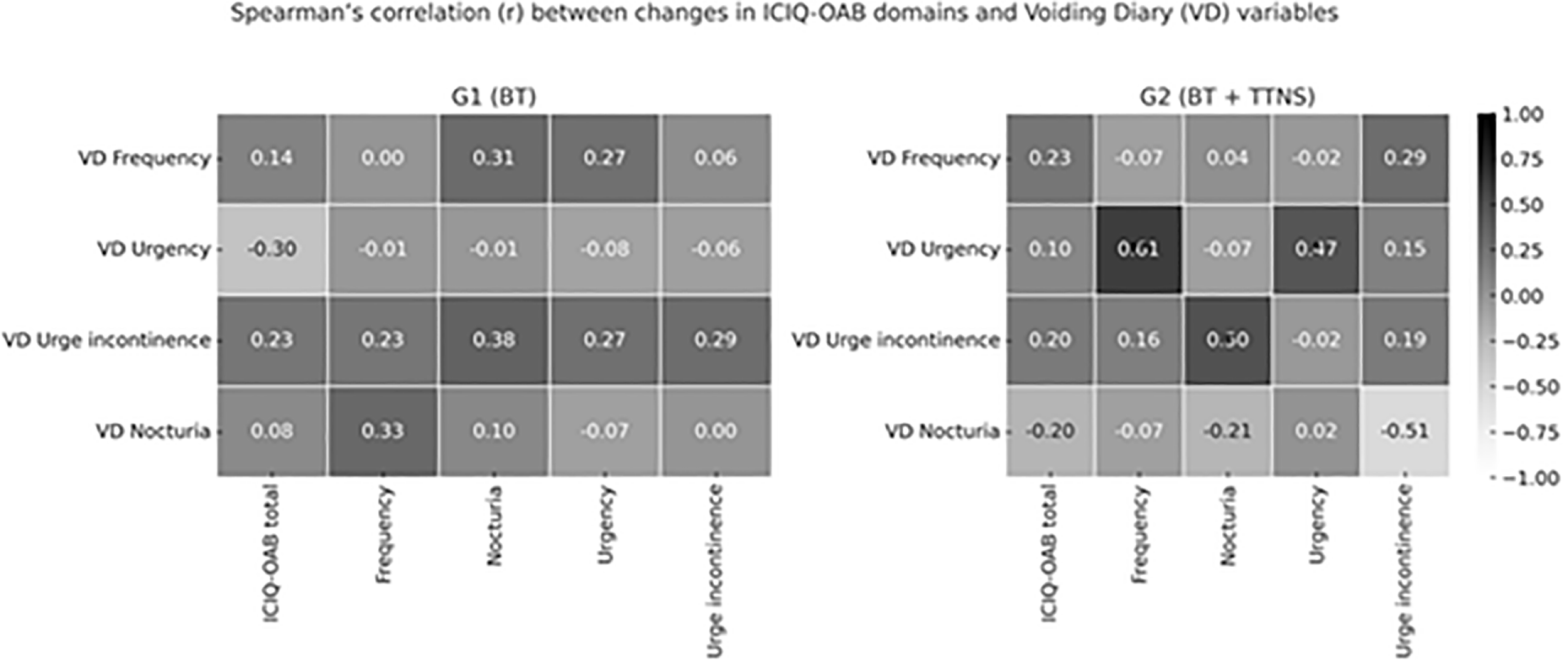
Spearman’s correlation between changes in ICIQ-OAB domains and Voiding Diary (VD) variables in G1 and G2 groups.

In Fig 2, we performed a correlation analysis between ICIQ-OAB scores and voiding diary (VD) measures in both groups, in order to examine the relationship between symptom evolution as assessed by changes (delta values) before and after the intervention. Delta variation (Δ) was defined as the difference between post-intervention and pre-intervention values (post – pre) for each outcome variable, allowing the assessment of individual changes over time. In G1, no significant correlation was found between changes measured by the ICIQ-OAB questionnaire and the voiding diary. A heatmap has been included to enhance the presentation (Fig 3). In G2, there was a strong positive correlation between the variation of episodes of voiding urgency with the discomfort of urinary frequency and between the delta variation of urgency urinary incontinence episodes with the degree of discomfort of nocturia (Table 4).

**Table 4:** Spearman’s correlation between changes in ICIQ-OAB degree of discomfort and Voiding Diary (VD) measures before and after intervention in G1 and G2 groups.

	Degree of discomfort				
	ICIQ	Frequency	Nocturia	Urgency	Urgency urinary incontinence
VD					
**G1**					
Frequency	0.14	0.00	0.31	0.27	0.06
Urgency	−0.30	−0.01	−0.01	−0.08	−0.06
Urgency urinary incontinence	0.23	0.23	0.38	0.27	0.29
Nocturia	0.08	0.33	0.10	−0.07	0.00
**G2**					
Frequency	0.23	−0.07	0.04	−0.02	0.29
Urgency	0.10	0.61**	−0.07	0.47*	0.15
Urgency urinary incontinence	0.20	0.16	0.50*	−0.02	0.19
Nocturia	−0.20	−0.07	−0.21	0.02	−0.51

*p < 0.05; **p < 0.01.

VD: voiding diary; G1: Behavior therapy (BT); G2: BT + Transcutaneous tibial nerve stimulations (TTNS).

It was performed on the correlation of ICIQ-OAB data with VD data in the two groups to observe the correlation between the symptoms’ evolution according to delta variation. In G1, there was no correlation between the variation of symptoms given by the instruments. In G2, there was a strong positive correlation between the variation of episodes of voiding urgency with the discomfort of urinary frequency and between the delta variation of urgency urinary incontinence episodes with the degree of discomfort of nocturia (Table 4).

No adverse events or complications related to the transcutaneous tibial nerve stimulation (TTNS) procedure were observed. All participants in both groups completed the study protocol without dropout.

## Discussion

This study provides innovative insights into the management of overactive bladder (OAB) in older women by directly comparing behavioral therapy (BT) alone with the combination of BT and transcutaneous tibial nerve stimulation (TTNS). Although TTNS has been evaluated in different contexts, few studies have focused specifically on its short-term effects in elderly women, a population often underrepresented in clinical research despite the high prevalence of OAB in this group. Furthermore, the use of the ICIQ-OAB questionnaire alongside a simplified, pictogram-based voiding diary represents a novel methodological approach, facilitating patient comprehension and adherence to data collection. By demonstrating that the addition of TTNS enhances symptom relief—particularly in urgency and nocturia—beyond that achieved by BT alone, this trial contributes unique evidence to support the feasibility, acceptability, and potential clinical utility of TTNS as a non-invasive adjunctive therapy in geriatric populations.

Behavioral therapy (BT) is recommended as first-line therapy in the Guidelines for OAB in adults [11,20]. The present study showed positive results with BT, a finding that corroborates to current evidence [21,22]. However, the combination of TTNS and BT resulted in higher gains in symptom improvement and QOL in older women when compared to BT alone. Schreiner et al. suggested that TTNS could be used as a first-line conservative therapy along with bladder reeducation and Kegel exercises in older women with urgency urinary incontinence due to reduced symptoms, improvement in QOL, and absence of adverse effects [14].

Voiding urgency was the symptom of greater discomfort in both groups at the beginning of treatment. After the intervention, there was a reduction in the discomfort of symptoms in both groups, however, with a significant difference only in the G2 intragroup analysis (p = 0.002). Other authors also found similar results in which urgency was the symptom of the greatest associated discomfort among men and women of different ages [22–25]). However, symptoms are more uncomfortable in females [2], and symptom severity is significantly associated with a reduction in patients’ QOL [26]. The studies also observed that the discomfort and the episodes of urgency increased with age [24,25]. Ridder et al. observed an increase of 34.8% of urgency in women between 40 and 50 years to 68.2% at the age above 80 years old [24]. In a study at a population level, urgency is the most prevalent symptom capable of generating moderate discomfort in patients (7.9%), followed by stress urinary incontinence (SUI) (6.5%) and nocturia (6%) [27].

The total score of the ICIQ-OAB questionnaire significantly decreased in G1 and G2, demonstrating improvement in the QOL of such women. In the intragroup comparison, G2 obtained a bigger score reduction of the questionnaire. Other studies corroborate the results, despite using questionnaires different from those applied in the present study. A study with 50 older women compared transcutaneous electrostimulation in tibial with parasacral. Both groups showed a reduction in the ICIQ-OAB score, meaning a reduction in the impact of symptoms on QOL after 8 intervention sessions. However, the variation in improvement represented by the reduction in the score of the previously mentioned questionnaire and the effect size of the therapy was higher in the group that received the TTNS [28]. Besides, TTNS was able to maintain symptom improvement and QOL for 6 months in women with OAB, who had already undergone treatment with percutaneous tibial nerve stimulation [29].

Among the studies that conducted methodologies similar to ours, with the use of TTNS, few used the ICIQ-OAB questionnaire to evaluate the intervention in the elderly with OAB [28,30]. Souto et al. used ICIQ-OAB to compare the effects of TTNS with oxybutynin in women with OAB and observed improvement in symptom reduction [31].

In the present study, we opted to apply a simplified VD and with the symptoms represented in the form of a pictogram to facilitate the participants’ understanding during its completion. In the VD’s information analysis, the G1 presented an episode reduction of urgency urinary incontinence and nocturia, but without a significant difference, and still, a slight increase of the episodes of urinary frequency and urinary urgency. In the G2, the variable of urgency urinary incontinence and nocturia had a significant reduction (p = 0.01 and p = 0.007, respectively). However, the urinary frequency and the urgency episodes increased slightly, but without a difference between groups.

The reduction of nocturia episodes occurred in both groups, but only G2 had a significant reduction. Both groups presented a reduction in symptom discomfort (G1 -p = 0.01; G2 p < 0.004). It demonstrates that behavior orientations’ changes, isolated or associated with electrostimulation, can reduce episodes and their degree of discomfort in older women. Furthermore, when expanding the results, it can reduce the risk of serious consequences and the personal and economic burdens involved.

It is important to highlight the distinction between statistical significance and clinical relevance when interpreting the findings of the present study. Although not all symptom domains demonstrated statistically significant changes, the magnitude and direction of the observed improvements — particularly in urgency and nocturia — suggest clinically meaningful benefits for older women with OAB. Even modest reductions in symptom frequency or discomfort can translate into substantial improvements in quality of life, independence, and social participation in this population. Statistical significance is influenced by sample size and study power, which were limited in our trial; however, the clinical impact of symptom reduction, corroborated by validated patient-reported outcomes, reinforces the practical value of the intervention. Therefore, the results should be interpreted not only through a statistical lens but also considering their real-world implications for patient care.

Although the precise mechanisms by which transcutaneous tibial nerve stimulation (TTNS) improves OAB symptoms are not yet fully understood, several hypotheses have been proposed. The tibial nerve shares roots with the sacral plexus (L4–S3), which innervates the bladder; thus, tibial stimulation may modulate the sacral micturition reflex [32]. This modulation is thought to occur through activation of somatic afferents that inhibit abnormal bladder sensory signaling at the spinal level, thereby reducing urgency and detrusor overactivity [32]. Experimental and clinical studies also suggest that TTNS suppresses involuntary detrusor contractions, possibly via inhibitory interneurons, while promoting short-term plastic changes that extend beyond the stimulation period [33,34]. Furthermore, TTNS may help rebalance parasympathetic and sympathetic activity in the lower urinary tract, contributing to greater bladder stability [35]. Finally, it is recognized that stimulation parameters (e.g., frequency between 10–20 Hz) influence outcomes, which may explain some variability across studies [36,37]. Taken together, these mechanisms help explain the symptomatic improvements observed with TTNS, although further neurophysiological research is warranted to clarify its exact mode of action [38].

Although our study focused on older women without neurological comorbidities, evidence exists supporting tibial nerve stimulation for OAB in neurogenic populations. In multiple sclerosis, PTNS has been shown in RCTs to significantly improve urinary urgency, frequency, bladder compliance, and urodynamic parameters when compared to pelvic floor training alone [39]. A long-term audit demonstrated sustained reductions in voiding frequency, nocturia, and incontinence for up to 24 months [40]. In Parkinson’s disease, home-based TTNS trials have reported symptom improvements lasting up to 90 days [41], though other studies have shown only limited benefits depending on stimulation protocols [42]. Emerging protocols in spinal cord injury suggest that TTNS is feasible, well-tolerated, and may reduce overactive bladder episodes and medication needs [43]. Taken together, the neurogenic data suggest potential benefit and safety of tibial nerve stimulation across diverse chronic conditions.

Additionally, another issue is the lack of standardization of stimulation parameters, number of sessions, duration of treatment, and follow-up. There is still no evidence of intervention program protocols with higher efficacy established, nor if longer electrostimulation times are presenting better results. What is well-grounded in the literature is that low-frequency electric current is indicated for OAB [13]. Booth et al. observed in their review that all studies used a frequency between 10 and 20 Hz [13]. Corroborating the results of the study in which the frequency of 10 Hz was responsible for inhibiting bladder contractions and being less uncomfortable for the patient, and which was used in the intervention protocol of the present study.

An additional relevant finding of the present study was the absence of complications related to TTNS. This result reinforces previous evidence supporting the safety and tolerability of this non-invasive technique, which is particularly advantageous for older women who are often more vulnerable to adverse effects associated with pharmacological treatments.

The present study’s results are encouraging, but the outcomes observed were only in short follow-up period (1 month), which restricts the ability to draw conclusions about the long-term persistence of the observed benefits. Nevertheless, the short-term results are clinically relevant, demonstrating significant symptom reduction and improved quality of life in older women with idiopathic OAB. Importantly, these findings are consistent with previous studies reporting the efficacy of TTNS in the short term, reinforcing its role as a promising conservative approach. Future research with longer follow-up periods is needed to confirm the durability of these outcomes. It is unknown if over time there will be a need for new treatments or even a possible repetition of the proposed therapy. To perform this type of therapy, the patient needs to move to a specialized health center or clinic, receive home care or can handle the household equipment for treatment. These requirements may hinder treatment compliance, especially if the elderly have any mobility difficulties. Even though there were no withdrawals throughout the therapy, we believe that the duration of 4 weeks was fundamental for this to occur.

Since the studied group was only composed of older women, aged between 60 and 80 years old, it is unknown if this therapy behaves in women of other ages. Besides, we did not use sham therapy in the control group, as it could have reduced expectancy bias and strengthened the internal validity of the findings. Future studies should incorporate sham-controlled designs to enhance the robustness and validity of the findings. The therapeutic effect of group 2 (TTNS and BT) concerning group 1 can be attributed to the difference in the number of visits in each group. We know that groups that have more contact with such therapy receive more encouragement and feel more motivated for clinical improvement. It could have been avoided if we had the sham therapy in group 1.

Although the prevalence of OAB increases with age, few studies are being conducted with this population. More researchers must focus on this population, both female and male.

In the present study, the greatest added value of TTNS appeared to be in the reduction of urgency episodes, highlighting its potential targeted effect on this particularly burdensome OAB symptom. However, our findings must be interpreted with caution, as the absence of a sham control group is a key limitation. Without a sham comparator, it is difficult to exclude the possibility that placebo effects or increased patient–therapist interaction contributed to the improvements observed. Future trials should therefore incorporate sham stimulation to better delineate the specific therapeutic contribution of TTNS.

Patients with neurological diseases were excluded from the present study because such conditions may cause or exacerbate lower urinary tract symptoms independently of overactive bladder (OAB). Including these patients could introduce significant confounding factors, making it difficult to isolate the specific effects of TTNS combined with behavioral therapy on idiopathic OAB symptoms. Unfortunately, we only asked the participant about the neurological disease, without a medical confirmation, we acknowledge this limitation.

## Conclusion

Both behavioral therapy (BT) and BT combined with transcutaneous tibial nerve stimulation (TTNS) improved overactive bladder symptoms and quality of life in older women, with greater benefits observed in the combined therapy group, particularly for urgency and nocturia. The intervention was safe and well tolerated, but results should be interpreted cautiously given the short follow-up and absence of a sham control. TTNS appears to be a valuable, non-invasive adjunct to conservative therapy, though larger, longer-term trials are needed to confirm these findings.

Our findings demonstrate that TTNS combined with behavioral therapy is a safe and effective short-term intervention for improving OAB symptoms and quality of life in older women without neurological diseases. Since patients with neurogenic OAB were excluded, the present conclusions apply specifically to idiopathic OAB, and future studies are needed to evaluate whether these results can be extended to women with neurological comorbidities.

## Supporting information

S1 File Anonymized_data. (DOCX)

S2 File CONSORT-2010-Checklist. (DOCX)

S3 File Data Availability_PLOSONE. (XLSX)
