# Correction: Randomized trial of transcutaneous tibial nerve stimulation to treat overactive bladder in older women

**DOI:** 10.1371/journal.pone.0353194

**Published:** 2026-07-06

**Authors:** Marianna Vale D’Alessandro Barbosa, Liana Barbaresco Gomide Matheus, Patrícia Azevedo Garcia, Júlia Shimohara Bradaschia, Marianne Lucena da Silva, Elaine Cristina Leite Pereira, Aline Teixeira Alves

The Funding statement for this article is incorrect. The correct Funding statement is as follows: We acknowledge the financial support from Edital nº 001/2026 DPI/BCE/UnB.
