# Supplementary material for: Randomized trial of transcutaneous tibial nerve stimulation to treat overactive bladder in older women
Source: PLoS One. 2026 Apr 6;21(4):e0322508. doi: 10.1371/journal.pone.0322508 (PMC13052882; doi:10.1371/journal.pone.0322508)
Supplement: S1 File — (DOCX) [file pone.0322508.s001.docx]

**Research Project**

**COMPARATIVE ANALYSIS OF THREE ELECTROSTIMUALIZATION PROTOCOLS IN THE TREATMENT OF OVERACTIVE BLADDER SYNDROME IN ELDERLY WOMEN: RANDOMIZED CLINICAL TRIAL**

Title: Comparative analysis of three types of electrostimulation in the treatment of Overactive Bladder Syndrome in elderly women: randomized clinical trial

1. **Research ethics committee (Yes / No):**

Protocoled: Not Accepted:

1. **What is the main hypothesis of the project (What answer do you want to get?)**

electrostimulation methods used in the treatment of Overactive Bladder Syndrome.

**3. What method was applied? (two lines)**

The method applied is electrostimulation transcutaneous posterior tibial nerve electrical stimulation transcutaneous parasacral and transvaginal electrostimulation

**4. Are there any publications from the group on the method used?**

The methods to be used are the same as those employed in most studies that use electrostimulation of the posterior tibial nerve, parasacral and transvaginal. However, there is no study that compares the three treatment modalities with a control group.

**5. Casuistry:**

**- Who:** women receiving care at a primary healthcare center in a metropolitan region of Brazil, referred from a public elderly health program.

- **How many:** all those who agree to participate in the project, with a minimum of 120 patients.

**6. Proposal for analysis (comparison, correlations, adjusted or multivariate analyses)**

Patients will be randomized into four groups. The first group will receive electrostimulation treatment of the posterior tibial nerve plus guidance ( urotherapy ), the second group will receive electrostimulation treatment parasacral electrostimulation plus urotherapy , the third group will receive transvaginal electrostimulation plus urotherapy and the fourth and last group will receive only urotherapy . The groups will be compared in relation to the improvement of the symptoms of Overactive Bladder Syndrome, through specific questionnaires, urine loss through the pad test and function of the pelvic floor muscles through bidigital evaluation. physiotherapeutic and electromyographic.

**7. Preliminary results**

There is none.

1. **Introduction**

Urinary incontinence (UI) is one of the main complaints of elderly patients, affecting between 15% and 30% of them, a third of whom require special care (RESNICK, 1998).

Szonyi *et al.* in 1995 reported that a typical cause of urinary incontinence (UI) in the elderly is urge urinary incontinence (UUI) or overactive bladder syndrome (OBS), characterized by urinary urgency associated or not with urge incontinence, pollakiuria and nocturia . Elderly people may be more susceptible due to side effects of medications, lack of necessary social or medical support, or the interaction of several pathologies that can lead to functional hyperactivity (GREEN, 1996).

Overactive Bladder Syndrome is a chronic condition that impacts quality of life, work productivity, social relationships, sexuality and physical activity. Several comorbidities are associated with OBS, such as fractures, lower urinary tract infection and depression (BROMWN, 2000). In addition, it requires changes in the patient's lifestyle.

Conservative treatment includes a controlled diet, scheduled fluid intake, medication, bladder training, biofeedback, pelvic floor exercises and electrostimulation. transcutaneous (MIKON, 2001).

In 2014, the American Medical Association defined electrostimulation as the second choice in the treatment of overactive bladder syndrome. The first, consisting of bladder training or behavioral guidelines (also called urotherapy) (QASEEM, 2014). However, in a randomized, controlled study that compared patients who underwent neuromodulation transcutaneous parasacral and the control group that underwent standard urotherapy, 31% of the group that received electrostimulation had improvement in symptoms and no patient in the control group had complete resolution of symptoms (LORDELO, 2010)

Antimuscarinic medications have also been used with a high rate of improvement in patients with lower urinary tract symptoms, but they are far from ideal treatment. The medications are usually used daily, and they carry side effects that can cause or worsen constipation and may lead to discontinuation of treatment.

Peripheral electrical stimulation has been widely used for the treatment of urinary disorders (McGUIRE *et al..* 1983, VODUSEK *et al.* . 1986, FALL *et al..* 1991, BRINDLEY 1994), including electrostimulation of the posterior tibia, vaginal electrostimulation and neuromodulation parasacral.

It is known that electrical stimulation of the sacral roots, as well as the posterior tibial nerve, activates inhibitory reflexes through afferent stimuli from the pudendal nerve, where activation of sympathetic fibers occurs in the pelvic ganglia and in the detrusor muscle. It also generates central inhibition of motor efferents to the bladder and of pelvic and pudendal afferents from the bladder. Therefore, the effects are due to the establishment of inhibitory mechanisms, with normalization of the balance between adrenergic and cholinergic neurotransmitters (LINDSTROM, 1983).

In 2001, Govier *et al.,* and Van Balken *et al.,* used stimulation of the posterior tibial nerve to treat urge incontinence and increased urinary frequency. Amarenco *et al., 2003, determined the* urodynamic effects following acute stimulation of the posterior tibial nerve in patients with symptoms of urge incontinence and frequency secondary to detrusor overactivity, with significant results for maximum cystometric capacity and first urge void volume. A recent study shows that there is no difference between stimulation of the posterior tibial nerve and neuromodulation. parasacral in children (BARROSO, 2013) however there are no studies in the adult population that compare treatment methods.

Key words: overactive urinary bladder, aged, stimulation, neuromodulation,

Keywords: overactive urinary bladder, elderly, electrostimulation, neuromodulation,

**2. Objectives:**

**2.1. General objective**

Analyze the effect of electrostimulation transcutaneous posterior tibial electrostimulation transcutaneous parasacral and transvaginal electrostimulation in the treatment of Overactive Bladder Syndrome through a quality of life questionnaire, symptom questionnaires and voiding diary.

**2.2. Secondary objective**

- Assess the socio-demographic aspects of the population studied;

- Correlate symptoms with quality of life using the ICIQ-SF

urinary symptoms: pollakiuria, nocturia, urinary urgency and urge incontinence through the urinary diary.

- Analyze the function of the pelvic floor muscles through electromyography and evaluate bidigital

urination habits

-Analyze evacuation habits through specific questionnaires.

- Analyze urinary losses through Pad-test

- Compare the three types of protocol and urotherapy

-Analyze the patients' state of anxiety and depression before and after treatment.

- Long-term reassessment of patients (1 year after treatment).

- Know the comfort level of each type of current on an analog scale of 0-10

**3. Methods:**

Randomized clinical trial study.

The study was conducted between 2015 and 2019.

Women aged 60 to 80 years old receiving care at a primary healthcare center in a metropolitan region of Brazil, referred from a public elderly health program, were included. Patients referred to this program were assessed according to the research group’s care protocol. Those who met the inclusion criteria were invited to participate, and those who agreed signed an informed consent form (Appendix I). The sample consisted of at least 120 participants.

Patients will be excluded if: they have stress urinary incontinence, have used medication to treat overactive bladder in the last six months, have any previous neurological disease, have a cardiac pacemaker, have a lower urinary tract infection, vaginal infection, vaginal discharge or any type of vaginal redness that characterizes pre-existing infection or dermatitis, have pain when urinating and/or suprapubic pain, have hematuria with sterile urine (suspected bladder carcinoma in situ ).

**3.1) Evaluations**

All assessments were carried out individually in a private clinical setting at a primary healthcare center in a metropolitan region of Brazil, ensuring participant privacy.

**3.1.1) Diagnosis of Overactive Bladder Syndrome and assessment of Overactive Bladder Syndrome symptoms:**

**3.1.1.1) ICIQ-OAB (International *Consultation on Incontinence Questionnaire Overactive Bladder)***

To diagnose and evaluate the symptoms of Overactive Bladder Syndrome, a specific questionnaire will be applied, the ICIQ – OAB *International Consultation on Incontinence Questionnaire Overactive Bladder* (TAMANINI, 2004). The ICIQ-OAB is a brief questionnaire with high psychometric capacity to specifically assess overactive bladder. It comes from the ICS ICIQ class, is fully validated and is capable of providing a measure to assess the impact of symptoms of urinary frequency, urgency, nocturia and incontinence.

**Voiding diary and voiding habits**

Another form of assessment will be the urinary diary. A urinary diary will be given, in which the patient must note the frequency urinary incontinence, episodes of urgency, episodes of urge incontinence and nocturia for three consecutive days. The patient should fill out the diary before and after treatment.

**3.1.1.3) Electric current comfort scale**

The patient must select the level of discomfort on a Visual Analogue Scale (0-10), with 0 meaning no discomfort and 10 meaning uncomfortable.

**3.1.1.4) 1-hour pad test**

Urinary losses will be assessed using a pad test. The pad test is a non-invasive test that quantifies urinary losses. The following protocol, already described in the literature, will be used: a) weigh the pad using a high-precision scale; b) ask the patient to drink 500 ml of water; c) ask her to sit for 30 minutes ; d) ask her to walk for 10 minutes ; e) ask her to sit and stand up from a regular chair 10 times; f) ask her to cough 10 times ; e) ask her to run/jog in the same place for 1 minute ; g) ask her to pick up an object from the floor 5 times ; h) ask her to wash her hands for 1 minute; h) then, the pad will be weighed again and the urine loss result given in grams.

**Urogynecological evaluation**

Before the collections begin, patients will be evaluated by a urogynecologist to assess genital dystopias, as well as to exclude any dermatitis, vaginal lesions, urinary and/or vaginal infections.

**3.1.1.6) Bidigital assessment and electromyographic assessment of the pelvic floor**

After investigating the complaints, a functional assessment of the pelvic floor muscles will be performed. The pelvic floor assessment is performed bidigitally by a physiotherapist, specialized in Pelvic Floor Rehabilitation , using the Oxford scale that grades perineal function into 6 degrees: 0: absence of muscular response from the muscles perineal ; 1: outline of unsustained muscle contraction; 2: presence of low intensity contraction, but sustained; 3: moderate contraction, felt as an increase in intravaginal pressure that compresses the examiner's fingers with a small cranial elevation of the vaginal wall; 4: satisfactory contraction, which squeezes the examiner's fingers with elevation of the vaginal wall towards the pubic symphysis; 5 strong contraction: firm grasp of the examiner's fingers with a positive movement towards the pubic symphysis

electromyographic evaluation follows the same idea as the functional evaluation of gait pelvic. However, a surface electrode ( Medcare ) will be used in the tendon center of the Perineum. The patient will be asked to contract the strongest possible MAP (maximum peak pressure) and sustain the contraction for as long as possible (endurance). The results will be given in mV.

**3.1.1.7) Anxiety assessment**

Beck Anxiety Inventory was created by Dr. Aaron Beck and is a self-report questionnaire with 21 multiple-choice questions used to measure the severity of an individual's anxiety and how they have been feeling over the past week. Scores of 0-7 are considered no anxiety, 8-15 is mild anxiety, 16-25 is moderate anxiety, and 26-63 is severe anxiety.

**3.1.1.7) Assessment of depression**

The Geriatric Depression Scale is one of the most frequently used instruments for screening depression in the elderly. Described in English, the original scale has 30 items and was developed especially for screening mood disorders in the elderly. The EGD, with 15 items, is a short scale of the original version and was developed by Sheikh & Yesavage (1986) from the items that most strongly correlated with the diagnosis of depression. Individuals who score 5/6 have a diagnosis of Possible Depression.

**Wexner 's assessment of bowel habits , constipation and incontinence**

patients' evacuation habits will also be assessed, such as weekly evacuation frequency , use of a bladder, sensation of incomplete emptying, use of the correct position to go to the bathroom, and delay in the urge to evacuate .

Wexner Constipation Questionnaires and Wexner Fecal Incontinence Questionnaire .

**3.2) Treatment**

**3.2.1) Behavioral guidelines**

All patients will receive behavioral guidance regarding Overactive Bladder Syndrome:

a) Correct way to use the toilet: always sitting, with your legs apart, body facing forward, elbows resting on your knees and using a footrest to keep your hips flexed above 90 degrees.

b) Scheduled urination: Patients should try to postpone urination as much as possible, trying to reach an interval of every 2 hours.

c) Avoid eating foods and drinks that irritate the bladder, such as caffeine, citrus fruits, and pepper.

**3.2.2) Electrostimulation transcutaneous tibialis posterior**

Electrostimulation​ transcutaneous in the posterior tibial nerve will be performed using the DUALPEX 961 ^® device^ – Quark brand.

The protocol used will be that of Amarenco *et al.,* 2003, in which the surface electrodes will be positioned with gel, one in the region posterior to the medial malleolus and the other 10 cm above. The correct position of the medial malleolus electrode will be determined by visualizing rhythmic flexions of the toes during stimulation with a frequency of 1 Hz and a pulse width of 200 µs . After observing the flexion of the toes, the frequency will be increased to 10 Hz. The intensity level will be adapted to the patient's sensory threshold. The patient should not feel any pain or discomfort during stimulation. Therefore, the intensity will become an individual parameter and may vary with each session. The session time will be 30 minutes and the frequency will be twice a week for 4 weeks, with an interval of at least 24 hours between sessions, for a total of 8 sessions.

**3.2.3) Electrostimulation parasacral transcutaneous**

Electrostimulation​ parascral transcutaneous will be performed using the DUALPEX 961 ^® device^ – Quark brand.

The surface electrodes will be positioned with gel, symmetrically in the parasacral region, under the posterior superior iliac spines . A frequency of 10 Hz and a pulse width of 700 µs will be used . The intensity level will be adjusted to the patient's sensory threshold. The patient should not feel any pain or discomfort during stimulation. Therefore, the intensity will become an individual parameter and may vary with each session. The session time will be 30 minutes and the frequency will be twice a week for 4 weeks, with an interval of at least 24 hours between sessions, for a total of 8 sessions.

**3.2.4) Transvaginal electrostimulation**

electrostimulation will be performed using the DUALPEX 961 ^® device^ – Quark brand.

The surface electrodes will be positioned inside the patient's vagina. A frequency of 10 Hz and a pulse width of 200 µs will be used . The intensity level will be adjusted to the patient's sensory threshold. The patient should not feel any pain or discomfort during stimulation. Therefore, the intensity will become an individual parameter and may vary with each session. The session will last 30 minutes and the frequency will be twice a week for 4 weeks, with an interval of at least 24 hours between sessions, for a total of 8 sessions.

**3.3) Statistical analysis**

Data were analyzed using both parametric and non-parametric tests, according to data distribution. Normality was assessed prior to test selection.

A significance level of 5% (p < 0.05) was adopted. Differences between groups were considered statistically significant when p-values were less than 0.05.

**4) Risks and benefits**

There are controlled risks associated with electrostimulation in the treatment of overactive bladder. According to studies, the current can cause physiological hyperemia of the skin, skin irritation, and occasional pain. Even so, these are reversible and non-severe adverse events ( Schreiner , 2013). Other studies demonstrate that electrostimulation is safe and effective (Smith, 1996) and is not responsible for any changes in the vaginal mucosa when used in this modality ( Rett , 2014). The intensity of the current is set at the patient's sensitive threshold, without pain. However, if for some reason the patient feels discomfort, the therapy is suspended without any detriment to the treatment.

The patient will benefit from free treatment for Overactive Bladder Syndrome.

**Work plan**

Activities to be developed:

- Carry out bibliographic review and update of the topic addressed

- Submit the project to the Research Ethics Committee

- Take courses in the Postgraduate program

- Analyze and discuss the collected data

- Prepare the manuscript and submit it to the scientific journal

- Write the thesis

- Defend the thesis

**5). Schedule**

| Tasks | 2015 | 2016 | | | | 2017 | | | | 2018 | | | |  |
| --- | --- | --- | --- | --- | --- | --- | --- | --- | --- | --- | --- | --- | --- | --- |
|  |  | 1st^​^ Trim | 2nd^​^ Trim | 3rd^​^ Trim | 4th^​^ Trim | 1st^​^ Trim | 2nd^​^ Trim | 3rd^​^ Trim | 4th^​^ Trim | 1st^​^ Trim | 2nd^​^ Trim | 3rd^​^ Trim | 4th^​^ Trim | |
| Fulfillment of credits | x | x | x |  |  |  |  |  |  |  |  |  |  | |
| Literature review |  | x | x | x | x | x | x | x |  |  |  |  |  | |
| Ethics approval and trial registration |  |  |  |  | x |  |  |  |  |  |  |  |  | |
| Qualification |  |  |  |  |  | x |  |  |  |  |  |  |  | |
| Data collection |  |  |  |  | x | x | x | x |  |  |  |  |  | |
| Data analysis |  |  |  |  |  |  |  | x | x |  |  |  |  | |
| Final Draft |  |  |  |  |  |  |  |  |  | x | x | x |  | |
| Writing/Publishing |  |  |  |  |  |  |  |  |  | x | x |  |  | |
| Article |  |  |  |  |  |  |  |  |  | x | x |  |  | |
| Defense |  |  |  |  |  |  |  |  |  |  |  |  | x | |

**6) Financial budget**

This study received no specific financial support. All materials and equipment were provided by the researcher. The study did not receive funding from any external agency.**7)**

**References**

AMARENCO, G., ISMAEL, SS, EVEN-SCHNEIDER, A., RAIBAUT, P., DEMAILLE-WLODYKA, S., PARRATTE, B. AND KERDRAON, J.: Urodynamic effects of acute transcutaneous posterior tibial nerve stimulation in overactive bladder. J Urol , 169: 2210, 2003.

BARROSO U JR. VITERBO W, BITTENCOURT J, FARIAS T, LORDELO P. Posterior tibial nerve stimulaton you parasacral transcutaneous neuromodulation for overactive bladder in children . U Urol. 2013 Aug : 190 (2): 673-7

BRINDLEY, GS The first 500 patients with sacral anterior root stimulator implants: general description. Paraplegia, 32: 795, 1994.

Brown JS, McGhan WF and Chokroverty S: Comorbidities associated with overactive bladder. Am J Managed Care 2000 ; 6:S574.

GREEN MF: Old people and disorders of incontinence, in Maldestam D ( ed ): Incontinence and its Management, ed 2. Dover, NH, Croom Helm, 1986.

GOVIER, FE, LITWILLER, S., NITTI, V., KREDER, KJ and ROSENBLATT, P.: Percutaneous afferent neuromodulation for the refractory overactive bladder: results of a multicenter study. J Urol , 165: 1193, 2001.

FALL, M. AND LINDSTROM, S.: Electrical stimulation. A physiological approach to the treatment of urinary incontinence. Urol Clin North Am, 18: 393, 1991.

LINDSTROM S. The neurophysiological basis of bladder inhibition in response to intravaginal electrical stimulation. J Urol 1983; 18:393-407.

LORDELO P, TELES A, VEIGA ML. Transcutaneous electrical nerve Stimulation in children with overactive bladder : a randomized clinical trial . J Urol 2010; 184: 683.

MCGUIRE, EJ , SHIN -CHUN, Z., HORWINSKI, ER AND LYTTON, B.: Treatment of motor and sensory detrusor instability by electrical stimulation. J Urol , 129: 78, 1983.

Milsom I, Abrams P, Cardozo L , et al. How widespread are the symptoms of an overactive bladder and how are they managed? A population-based prevalence study. BJU Int. 2001 ;87:760 -766.

QASEEM A, DALLAS P, FORCIEA MA,STARKEY M, DENBERG T, SHEKELLE P. Nonsurgical Management of Urinary Incontinence in Women . Ann Intern Med.161 (6) 429-440.

PAICK JS, KU JH, SHIN JW et AL. Significance of Pad test loss for the evaluation of women with urinary incontinence. Neurourology and Urodynamics 24:39-43 (2005)

RESNICK NM. Improving treatment of urinary incontinence. JAMA ;280:2034 -35, 1998.

SMITH J. Intravaginal stimulation: a randomized trial. The journal of urology; 155:127-130, 1996

SCHREINER L, dos SANTOS TG, de SOUSA AB et al. Electrical Stimulation for Urinary Incontinence in Women : A Systematic Review . Int. Braz. J. Urol. 39 (4): 454-464, 2013

Tamanini JTN, Dambros M, D'Ancona CAL, Palma PCRP, Netto NR. Validation of the “International Consultation on Incontinence Questionnaire - Short Form” (ICIQ-OAB) for Portuguese. Health Review Public 2004 ;38 (3):438-44

VAN BALKEN, MR, VANDONINCK, V., GISOLF, KWH, VERGUNST, H., KIEMENEY, LAL, DEBRUYE, F. M, J. et al.: Posterior tibial nerve stimulation as neuromodulative treatment of lower urinary tract dysfunction. J Urol , 166: 914, 2001.

VODUSEK, DB, LIGHT, JK AND LIBBY, JM .: Detrusor inhibition induced by stimulation of pudendal nerve afferents. Neurourol Urodyn , 5: 381, 1996.

**ANNEXES**

**FREE AND INFORMED CONSENT FORM - TCLE**

You are being invited to participate in the project: **Effects of treatment physiotherapy and surface electrostimulation in the treatment of Overactive Bladder Syndrome in elderly women** .

The objective of this research is: to analyze the effect of three types of surface electrostimulation protocol and urotherapy in the treatment of overactive bladder.

You will receive all necessary information before and during the research and we assure you that your name will not appear and that the strictest confidentiality will be maintained through the total omission of any information that could allow you to be identified.

In the first meeting, a physical examination (gynecological examination) will be performed to assess the strength of the pelvic floor muscles. The following questionnaires will also be administered: a preliminary questionnaire prepared by the authors, an ICIQ-OAB quality of life questionnaire on urinary symptoms, and a 3-day urinary diary to be completed at home. In the second and subsequent meetings, electrostimulation of the tibial nerve (in the foot), or parasacral (in the back), or intravaginal (in the vagina) will be applied. The treatment consists of 8 meetings, twice a week. All sessions will be scheduled in advance and performed in a private clinical setting.

Please be advised that you may refuse to answer (or participate in any procedure) any question that may cause you embarrassment, and you may withdraw from the research at any time without any harm to you. Your participation is voluntary, that is, there is no payment for your collaboration.

The results of this research may be published in scientific journals. All data will be stored securely by the research team for a minimum of five years.

This study was approved by a Research Ethics Committee. All participants provided written informed consent prior to participation.

This document was prepared in two copies; one will remain with the researcher in charge and the other with the research subject.

______________________________________________

Name / signature

____________________________________________

Responsible Researcher

Name and signature
